# Experiences of families living with Nyaope users in Tshwane, Gauteng province

**DOI:** 10.4102/hsag.v29i0.2338

**Published:** 2024-03-29

**Authors:** Dorcas Nene, Florah Mkhonto, Kebogile E. Mokwena

**Affiliations:** 1 Department of Nursing Sciences, School of Healthcare Sciences, Sefako Makgatho Health Sciences University, Pretoria, South Africa; 2 Department of Public Health, School of Healthcare Sciences, Sefako Makgatho Health Sciences University, Pretoria, South Africa

**Keywords:** nyaope, qualitative research, Tshwane families, family support, coping strategies, experiences and nyaope

## Abstract

**Background:**

Nyaope is one of the commonly used drugs in many low socio-economic communities in South Africa. Because of its highly addictive properties, the vast majority of users are not able to quit, which results in long-term difficulties for their families.

**Aim:**

The aim of this study was to explore and describe the experiences of families living with nyaope users in a township in Tshwane Metropolitan Municipality.

**Setting:**

Data were collected at the Social Development Centre, which serves a variety of social needs of families, including the various needs of families whose problems emanate from nyaope use. The family members were recruited from the registry of the centre.

**Methods:**

A qualitative design and in-depth interviews were used to collect data from a sample of family members who were purposively sampled. NVIvo 12 was used for thematic analysis of the data.

**Results:**

Three major themes emerged from the data, these being, consequences of nyaope use on the family, family interventions, and coping strategies.

**Conclusion:**

Although the families have devised interventions and developed coping strategies, nyaope use remains a serious mental health challenge in affected families.

**Contribution:**

The study highlighted the negative impact of nyaope on the social and mental health of the families. The assistance offered at Social Development is broad and general for substance abuse, but does little to mitigate the complex difficulties brought about by nyaope use.

## Introduction

The global and local increase in the use of psychoactive substances has been well documented (Tran et al. 2019). Because of consequential behaviours of substance abuse, their negative impact and challenges that also affect the family members has been well documented (Carvalho et al. 2019; Madiga & Mokwena 2022), and such impacts include mental as well as physical manifestations (Modis, Mokgaola & Sehularo 2021), which suggests that efforts to address the societal problems of substance abuse should not be limited to the user, but the family members as well.

Novel Psychoactive Substances (NPSs), also known as cocktail or designer drugs, are a new category that is gaining momentum in various parts of the world, including South Africa. Because they consist of various compounds, it is difficult to control them under the international drug treaties NPSs are not controlled under the international drug treaties (Collins 2017).

The NPSs present multifold challenges that include legislative challenges for governments (King & Kicman 2011; Smith & Garlich 2013). Governments also struggle to develop appropriate policies regarding their detection, identification, monitoring and responding to their use (Griffiths, Evans-Brown & Sedefov 2013). Often, there are inadequate data on their metabolism, toxicity and scheduling (Stojanovska 2013). Additionally, it is difficult to countermeasure the distribution of NPSs because of their variety, the continuous introduction of new compounds and linguistic diversity in regions, nationalities and communities. Therefore, the effects of many NPS drugs are not known in details, but their health effects include toxicity of various systems of the body and brain dysfunction (Dargan & Wood 2013; Dawson & Moffat 2012; Wood, Greene & Dargan 2011).

Nyaope is the main NPS in South Africa, and it fits the category of NPSs because although not controlled under international drug treaties, it is addictive and has the same psychoactive effects as other chemically related, controlled substances. It is cheap, widely used and readily available. The effects of nyaope are devastating on the person using it and several publications have reported about its severe negative impacts on the family members of the users (Masombuka & Qalinge 2020; Nkosi 2017).

Nyaope is a cocktail or NPS that continues to be commonly used in many black townships of South Africa since the early 2000s. Its composition varies across samples, with the most common components being cannabis, heroin and anti-retrovirals, (Mthembi, Mwenesongole & Cole, 2018). It is also known as ‘sugars’ (Vahed 2015) and ‘whoonga’ (Shembe 2013). Although its prevalence has not been determined, it remains the most commonly used illicit drug in black townships because it is easily accessible. Nyaope was classified as illegal in March of 2014 (Mokwena 2015), but this change in legislation has not assisted in reducing its distribution, sale and use over the years. Users can be easily identified as they assemble in groups in street corners, taxi ranks and parks, have very poor hygiene with slow speech and movement. Nyaope users often become so preoccupied with obtaining the next ‘fix’ that they are unaware of what is going on around them. Consequently, they neglect the needs of their family members leading to the collapse of the family as a unit (Mathibela 2017).

A typical common unbecoming behaviour of nyaope users is stealing any items they can lay their hands on, which they sell to get money to feed their habits. This habit of stealing is the main contributory factor to anxiety and even depression among family members who are often the first victims of such thefts. Because rehabilitation outcomes are poor because of high relapse rates, nyaope use leaves families with feelings of helplessness, disappointment, and frustration (Schultz & Alpaslan 2016). Nyaope does not only affect the user, but it is also associated with mental health challenges for significant others of the users, such as parents, siblings, partners, and other extended family members (Mokwena & Makuwerere 2021) and specifically depression (Madiga & Mokwena 2022).

The high rates of nyaope use have become a major concern for South African communities and impact negatively on the increasing number of users, their families and broader communities. Families are exposed to the continuous unbecoming behaviour of the user, which often attracts retaliation by community members, and thus safety concerns for the users and their families. Other concerns relate to the desperation of seeking, and often failing to get appropriate help relating to rehabilitation, as well as the continuous thefts occurring in their homes. However, the most critical challenge of nyaope use is the very high relapse rate even after rehabilitation, which has resulted in people who started using the drug in their twenties, still continuing its use in their late forties, despite many admissions at rehabilitation programmes (Mokwena & Fernandes 2015; Mokwena & Morojele 2014). Although the Department of Social Development provides support families with various social problems, including those related to nyaope use, anecdotal evidence suggests that many people find it difficult to deal with the consequences of nyaope use within their families. Most of the studies on nyaope use focus on the users, but there are few who focus on the impact of nyaope use on the families who live with the users. The purpose of this study was to explore the experiences of families who live with nyaope users in a Tshwane township.

## Research methods and design

### Study design

A qualitative, exploratory and descriptive design was used for the study, and in-depth interviews were used to collect data.

### Study setting

The study was conducted in a township in Tshwane, where nyaope use is rife. Within the township, data were collected at the Social Development Centre, which serves the social needs of families, including the needs of families whose problems emanate from nyaope use within their homes, such as attempts to get help for their relatives who use psychoactive substances.

### Population

The population consisted of family members who live with a nyaope user. The family members could be any nuclear or extended member of the family, including mothers, fathers, grandparents, uncles, aunts and siblings.

### Recruitment

Participants were recruited from the Social Development Centre in the township. The clearance certificate from the Sefako Makgatho University Research Ethics Committee was used to negotiate for permission to conduct the study from the Gauteng Social Development Centre and that approval letter was presented to the local Social Development Centre as support for request to conduct the study. The social worker within the centre was requested to identify individuals who contact the centre regarding problems related to nyaope use by members of their families. The individuals could be sisters, uncles, mothers or fathers of the nyaope user. The social worker would inform them about the study, and those who were interested were referred to the researcher for further details about the study and their potential participation.

### Sampling

Purposive sampling was used, and the inclusion criteria were family members who reached out for help at the Social Development Centre to get help for the nyaope users, were 18 years of age or older, and were willing to participate in the study.

### Sample size

The sample size of 15 participants was determined by data saturation, which was the stage in which additional interviews were no longer yielding new information.

### Data-collection tools

A researcher-developed in-depth interview guide was used and a quantitative questionnaire was used to collect socio-demographic data.

### Data-collection process

Data were collected in English, Setswana and Sepedi, according to the preference of the participant. On the day of data collection, the participant was invited into the interview room. The purpose of the study was explained, and he or she was given an opportunity to ask questions or seek clarifications about the study and their participation. When all the questions were answered, they were asked to provide written informed consent, which was followed by the administration of the socio-demographic questionnaire and the interview guide, which included probes and follow-up questions. The interview was digitally recorded. The necessary precautions related to the COVID-19 pandemic were applied in accordance with the Sefako Makgatho Health Sciences University Research Ethics Committee recommendations.

### Data analysis

The audio data from the digital recorder were transcribed verbatim, translated to English, typed into Word, and uploaded into NVIvo 12 for thematic analysis. The analysis started with reading the first two transcripts several times to familiarise the researcher with the data. The use of similar words, phrases, and expressions were identified, grouped together as codes or themes and assigned a specific meaning and/or definition. These definitions were the first version of the codebook, which was amended as new codes were developed, merged, or dropped. The codes and/or themes were then applied to all the transcripts using the NVIvo 12 software. The themes were used to develop the narrative of the findings of the study. The supervisor and co-supervisor of the study supervised the coding process.

### Trustworthiness

The trustworthiness of the study was enhanced by the use of NVIvo software, which enhanced consistency in the development of the themes, including the naming and definition of the emerging themes. The use of verbatim statements demonstrated and supported the emergence of the themes, and the detailed description of the research processes enhanced the transferability and dependability of the study.

### Ethical considerations

The study proposal was approved by the Sefako Makgatho Health Sciences University Research Ethics Committee (SMUREC/H/03/2021:PG), and permission to conduct the study in the centre was granted by Gauteng Social Development. All the participants provided informed written consent, with all related principles. The interviews were conducted in a private space and all information obtained from the participants was kept confidential.

## Results

### Characteristics of the participants

The socio-demographics of the 15 participants are shown in Table 1.

**TABLE 1:** Socio-demographic profile of the participants.

Variables	*n*
Number of participants	**15**
**Age range of the participants**	
20–29 years	1
30–49 years	4
50–59 years	4
60–69 years	5
70–79 years	1
**Relationship to nyaope user**	
Sister	2
Mother	10
Father	2
Uncle	1
**Employment status**	
Employed	7
Unemployed	4
Pensioners	4
**Gender**	
Male	3
Female	12
**Marital status**	
Single	6
Married	5
Divorced	4

### Qualitative findings

Three major themes and 11 subthemes emerged from the study, as shown in Table 2.

**TABLE 2:** Themes and subthemes.

Theme		Sub-theme
1.	Consequences of nyaope use on the family	1.1 Family conflicts 1.2 Employment implication for family members 1.3 Financial implication for family members 1.4 Mental distress
2.	Family interventions	2.1 Contacted social worker 2.2 Beating up nyaope user 2.3 Chasing nyaope user away from home 2.4 Resorting to prophets or traditional healers
3.	Family coping strategies	3.1 Locking the nyaope user out of the house 3.2 Through prayer 3.3 Talking to people

#### Theme 1: Consequences of nyaope use on the family

This theme refers to the views of the participants regarding a variety of changes that have occurred because of their relative’s use of nyaope. A further three sub-themes emerged from this theme, as detailed further in the text.

**Subtheme 1.1: Family conflicts:** The use of nyaope by a member of the family often results in conflicts in the family, which includes conflicts among siblings, and even spouses as reflected in the quotes discussed further in the text:

‘My daughters blame me that I am spoiling him by allowing him to drive my car. My husband and I are always fighting.’ (48-year-old-mother of the user)

‘We are not in good terms; they (other children) don’t talk to me and my son.’ (56-year-old-mother of the user)

‘Her sister is not even talking to him due to his behaviour of using nyaope.’ (59-year-old mother of the user)

**Subtheme 1.2: Employment implication for family members:** This subtheme refers to negative impacts of nyaope use on the employability of the parents, whose work commitments are often compromised because they need to attend to various problems of their child who uses nyaope.

The family members, especially the parents, often miss work as they try to find the nyaope user when he or she goes missing:

‘That day he did not come back home. Following day, I did not go to work, I went back to the taxi rank, he was not there.’ (62-year-old father of the user)

They miss work as they attend to legal cases, which are many as the users frequently engage in illegal activities:

‘I must absent myself from work and attend his court days. I might lose my work as I am only working three days per week.’ (56-year-old mother of the user)

Even when at work, the parent is often distracted:

‘At work I was just worried about him, I did not concentrate properly. My work performance has degraded.’ (48-year-old mother of the user)

‘My mind is not 100% at work it is not easy to concentrate or fully function at work. It might affect my work performance.’ (52-year-old mother of the user)

Absence from work even results in potential and/or actual loss of employment:

‘I might lose my job as I can’t concentrate at times.’ (41-year-old sister of the user)

Attempts to stop the addiction process even lead to the parent resigning from work:

‘I even resigned from work as I was working different shifts, sometimes I was working day and sometimes night as I was worried that he will be an addict of nyaope.’ (49-year-old mother of the user)

**Subtheme 1.3: Financial implication for family members:** Nyaope use comes with financial burden because of various reasons. The responses from the participants indicated that the families preferred to give the user money and all that he needs to prevent him from stealing. Against their moral judgements, the participants continue to give the users money, reasoning that it is better than to let them steal from others, which results in perpetual draining of their resources:

‘Even now I am still giving him money to buy nyaope to avoid that he steals.’ (62-year-old-father of the user)

The unemployment status of the users, as well as their continuous demands continue to be a financial burden to the parents:

‘Financially he is not stable as he is not working, he depends on me as he knows the mother will not give him any money. He will ask for money for cigarettes, and I will just give him.’ (65-year-old-father of the user)

‘But when he asks money from me, I sometimes give him behind my husband’s back to avoid him stealing. I gave him money to buy things.’ (48-year-old-mother of the user)

The users also manipulate their parents by convincing them that the fix makes them feel ‘better’:

‘I gave him money to buy nyaope as he asked me that he will be better after getting a “fix.” He is really draining me financially and psychologically. I am struggling with him financially.’ (56-year-old-mother of the user)

They use a variety of reasons to justify their need for money from their parents:

‘He lost his ID again. He requested money again to re-apply for it again to register for this monthly R350.00 social relief grant of COVID-19.’ (57-year-old-mother of the user)

The manipulation includes motivation to retrieve stolen items that they sold:

‘After stealing and selling stolen items, he will come back to us asking for money to get back those items back.’ (41-year-old-sister of the user)

**Subtheme 1.4: Mental distress:** Participants indicated mental distress that results from the combination of continuous problems of living with a nyaope user:

‘To tell you the truth my sister, it is stressful. The other day I was so worried, he did not even sleep at home. I am fearing that what is going to happen to him if I pass on.’ (62-year-old-father of the user)

‘It is not easy to live with a nyaope user, is stressing and painful looking at your child suffering. I have a big burden on my shoulders, so it is very difficult to cope [sigh]. It is very difficult to cope.’ (49-year-old-mother of the user)

Previous painful experiences of other nyaope users in the family worsen the mental distress:

‘Eish it is painful to live with a nyaope user. What is stressing me worse, my younger sister passed on due to nyaope. I will wait for them to sleep then my tears would start to come out.’ (48-year-old-mother of the user)

#### Theme 2: Family interventions

This theme refers to the participants’ attempts to deal with nyaope use by their relative. Five subthemes emerged from their responses that will be discussed further in the text.

**Subtheme 2.1: Contacted social worker:** Social workers are common first call for assistance regarding rehabilitation opportunities. The participants are often advised to resort to social workers, with the hope that they will get the required assistance.

Family members often approach social workers for assistance with getting the nyaope user to a rehabilitation centre, as well as other matters relating to nyaope addiction and undesirable behaviours of the nyaope user as highlighted further in the text:

‘Before rehabilitation I accompanied him to the social worker to know the whole story and procedure for admission in a rehabilitation centre.’ (65-year-old-father of the user)

‘My son told me about the social worker dealing with substance abuse in that centre and that he assisted most of nyaope users. Then the very social worker took him to this other second rehabilitation centre.’ (70-year-old-mother of the user)

‘My mother phoned the number she got on the pamphlet, then they referred her to the social worker who assisted my brother to go for rehabilitation.’ (21-year-old-sister of the user)

**Subtheme 2.2: Beating up nyaope user:** Physical violence is commonly perpetrated against the nyaope user. This theme refers to the physical assault of the nyaope user by family members, which occurs easily because nyaope users are physically weak and can hardly fight back. These assaults occur for different violations of family norms as explained next:

‘His mother even beat him.’ (65-year-old-father of the user)

‘His father started to be curious and checked his bedroom window; the burglar was a little bit loose. Then he suspected that he went through the window. He was so angry he beat him until he was unconscious.’ (48-year-old-mother of the user)

‘My heart was sore when my brother beat my son for things which he never stole. He was throwing bottles to my son, he is that type of person that when he beats your child, he will throw at him whatever he comes across.’ (56-year-old-mother of the user)

**Subtheme 2.3: Chasing the nyaope user away from home:** After exhausting various solutions, family members often resort to chasing the user away from home, with the intention of removing the various problems that the user brings to the family:

‘Her mother ends up chasing him away from the house.’ (65-year-old-father of the user)

‘But now after his second relapse, the other sister said they must chase him away.’ (70-year-old-mother of the user)

‘Then I decided to chase him away. He went to stay with his mother.’ (62-year-old-father of the user)

**Subtheme 2.4: Resorting to prophets and traditional healers:** This subtheme refers to families resorting to spiritual and religious solutions, as they try to make sense of the situation, and to find a solution. Sometimes they explain the strong addiction and frequent relapse as witchcraft, which prompts them to seek a related solution:

‘Then his mother suspected that he has been bewitched. He took him to a prophet to help him to stop this usage of nyaope by giving him some treatment to take.’ (65-year-old-father of the user)

‘Yes, so far. I also took him to the traditional healer. I thought maybe it was witchcraft. His strange behaviour of acting like a mad person, made me suspicious that he has been bewitched.’ (45-year-old-mother of the user)

‘Two to three weeks ago, someone told my brother about a traditional healer who helps nyaope users. My brother took my youngest son and his to that healer.’ (52-year-old-mother of the user)

#### Theme 3: Family coping strategies

Participants explained different ways that they use to cope with the difficult situation of living with nyaope users. Four subthemes further emerged from their responses, as discussed further in the text.

**Subtheme 3.1: Locking the nyaope user out of the house:** Participants often resort to locking the nyaope user out of the house, to prevent him from stealing various items:

‘During the day if I went to do my piece jobs, I used to lock him outside the house, until I come back.’ (65-year-old-mother of the user)

‘It is not easy to stay with him you must lock the bedrooms so that he must not steal our belongings. We only left the kitchen and dining rooms for him, as he must eat and watch television.’ (45-year-old-mother of the user)

‘When we go out, we lock our bedrooms, as it is not safe to leave him alone in the house as he might steal from the bedrooms.’ (59-year-old-mother of the user)

**Subtheme 3.2: Through prayer:** Seeking divine interventions, through prayer, is often a coping strategy, because this is used for both spiritual and mental strengthening.

The family member’s use of nyaope is reported to draw the parent to God:

‘I can say, I am a very prayerful man, and since he started using this nyaope, I got closer to God. He made me to concentrate on the word and in prayer, sometimes I even fast for him, and ask God to help him to quit.’ (62-year-old-father of the user)

The prayer groups also serve as a social support system:

‘I also have a prayer group; we usually pray at 12 midnight and pray through WhatsApp group. It really helps me.’ (48-year-old-mother of the user)

‘I usually go to church so that they pray for me because they know my child’s problems.’ (49-year-old-mother of the user)

‘I have a prayer group at church, which we usually share some prayers, especially if one of us feels down.’ (56-year-old-mother of the user)

**Subtheme 3.3: Talking to other people:** This subtheme refers to the practice of sharing their problems with specific people, which brings some relief from stress as talking with other people about the nyaope problem seems to assist with coping:

‘So far, I am tolerating him, as me and her sisters we usually express or talk about it so far, we are coping.’ (70-year-old-mother of the user)

‘I feel better when I “ventilate” to my colleagues at work.’ (45-year-old-mother of the user)

‘I also communicate with my daughter or my friend after that I will feel much better.’ (62-year-old-mother of the user)

## Discussion

The three major themes, that is consequences of nyaope use on the family, the interventions that the family tried to resolve the problem, and the coping strategies that they developed to deal with nyaope use in their family settings summarise the experiences that the study purposed to explore. From these major themes, viable recommendations can be made.

The socio-demographics of the participants confirm that more women are affected by problems of nyaope use by their family members, especially children, who are mostly adults. In a related study, Madiga and Mokwena (2022) also reported that because more women care for nyaope users, they are more likely to develop associated mental disorders. The finding that nyaope use results in family conflicts, which includes negative impact on the relationship between the parents and other children, was previously reported by other studies (Mafa & Makhubele 2020; Makhawula 2018). This happens because other siblings may be of the view that their needs and concerns are often ignored or minimised, while parents focus on the crises of the substance abuser (Choate 2015). Physical fights and verbal arguments, which are directly related to the use of substances by a family member often occur among family members although they themselves do not use substances (Ngantweni 2018).

This study found that nyaope use affects the employability of the parent, which was also previously reported, that is the various problems of a family substance abuser compromise the parent’s coping ability at work (Choate 2015), as the affected families are required to adjust their lives, including working schedules, to respond to the needs of the substance abuser (Ngantweni 2018). The employability status of families is further affected as they have to deal with problems during working hours (Matheba 2020) and parents are often required to take time off from work and other responsibilities to focus on the problems that their teenagers engage in, such as attending a court hearing (Mathibela 2017).

The finding that family members experience mental stress has been previously reported by Nkosi (2017), that is that families living with the person dependent on substances experience emotional stress as they have to deal with the consequences of the dependence and/or actions of substance use. These emotional impacts have been specifically reported to be heightened for nyaope users (Madiga & Mokwena 2022; Mokwena & Makuwerere 2021), as nyaope dependence is a threat to lives of the users (Khumalo 2020)

This study found that the participants often resort to social workers as the first port of call, which is expected, as social workers are known to assist with a range of social problems, including access to rehabilitation programmes. Moreover, such social worker services are mostly available free of charge through non-governmental organisations (Ngantweni 2018). Moreover, in South Africa, parents yearn to be supported by the community and professionals such as social workers (Choate 2015; Mafa & Makhubele 2020), who can provide counselling as well as advice in cases of mental distress and hopelessness, which commonly occur in families who have a nyaope user.

Although there are established rehabilitation programmes for a variety of substances, often times such intervention programmes do not result in desired outcomes, which leave families needing more resources that will enable them to deal with their family members and bring in changes in their lives (Choate 2015). Moreover, nyaope is one of the substances in which the relapse rates are very high (Fernandes & Mokwena 2016; Mahlangu & Geyer 2018), and for which there is no evidence-based custom-developed rehabilitation programme. For this reason, there are few individuals who remain clean for a prolonged period of time, even after completing nyaope rehabilitation programmes. This reflects negatively on nyaope users and their families, who view their status as not changeable. The situation further requires ongoing reflection by stakeholders on substance abuse prevention programmes, which should be implemented to decrease the prevalence of nyaope use (Mohasoa 2018).

Families often try to implement their own interventions, and in this study, such reactions include violence perpetrated against the nyaope user, chasing the user away from home and resorting to prophets and traditional healers. Such actions are prompted by the desperation of the family members, especially as they experience repeated relapse after rehabilitation. Matheba, Kgadima and Mabelane (2021) report that common reactions of families include punishment, harsh discipline, and physical restraint towards the nyaope user, as they experience anger, frustration, and hopelessness.

In townships where nyaope use is rife, violence is often used by the community when the nyaope user is caught stealing (Khumalo 2020). Community members commonly resort to informal and illegal mechanisms, such as physical assaults, which often include *sjambokking* or beating with a whip (Matheba 2020). It is such reports of violence that result in family members developing mental distress because they know that their family member can be a victim of such assaults. The same concerns apply after the nyaope user is banished from home, as they are exposed to violence by members of communities. Schultz and Alpaslan (2016) reported that some families may avoid disciplining a nyaope user for fear of escalating his behaviour and they use avoidance and ignorance as a coping strategy.

The finding that some families resort to traditional healers to solve the problem of nyaope addiction was previously reported by Mohasoa (2018), who concluded that traditional healers become a viable option because traditional healers perceive themselves to be adequately trained to heal various illnesses, including spiritual illness, which include substance use addiction, in general, and nyaope use in particular. Ngobe (2015) further indicated that African traditional perspectives offer a parallel belief system about the origins of substance abuse and its treatment with herbal medications, which resonates with people with the same belief system. Spiritual convictions are easier for parents who are inclined to seek spiritual over professional interventions, especially when they perceive that nyaope users are possessed by evil spirits or affected by witchcraft (Mafa & Makhubele 2020).

Waini (2015) and Choate (2015) report that parents often give their teenagers money to buy and use substances, as a way of assisting them to avoid criminal records if they steal from other people, which was also a finding in this study. This adds the financial burden on families of nyaope users, with other costs being rehabilitation fees (Groenewald & Bhana 2015; Khumalo 2020) and the various items that are frequently stolen to be sold to feed the nyaope habit.

The stealing behaviour of nyaope users results in parents and siblings having to have their eyes on their belongings all the time, as well as keeping the house under lock and key when they are away (Ngantweni 2018), which was a measure of desperation reported in this study. This is consistent with the findings of Khumalo (2020), and it occurs because of the fear that the addicts would steal valuables from home (Fernandes & Mokwena 2016). Other measures include putting burglar bars on the windows, to prevent nyaope users to access the house or specific rooms (Mokwena 2016). This act indicates the severity of mental stress experienced continuously, which explains why family members of nyaope users are reported to have quantifiable and significant symptoms of depression (Madiga & Mokwena 2022).

The finding that coping strategies include prayer is consistent with that by Schultz and Alpaslan (2016), who reported that some families use prayer as their coping strategy, as this keeps their hope that users will change their substance use and refocus their lives. Although some faith-based organisations view psychoactive substance use as dishonourable and sinful, families still seek spiritual help in dealing with their family members’ substance use behaviours from the same organisations (Ngantweni 2018). After all, churches have the responsibility to respond to a variety of challenges, including those that are related to substance abuse in families, as well as to offer counselling for the affected people (Masombuka 2013); this explains the tendency of family members to access that resource. Additionally, the church offers support, which results in hope and courage (Waini 2015).

### Strengths of the study

A strength of the study was that the participants were recruited from a service centre, which assists residents with a range of social problems; thus their input was on specific problems that they had thought through.

### Limitations of the study

A minor limitation of this study is the fact that all of the participants played a parental role for the user, and the views of siblings and younger members of the families were not heard. This may have brought additional perspectives to living with a nyaope user.

### Recommendations

The recommendations that emerge from the results of this study are the following:

1. That the Department of Social Development creates a section that is specific to provide mental and social support for families of substance abusers, with a focus on nyaope. This is because nyaope addiction presents with unique challenges that are not necessarily similar to the use of other substances.
2. That a professional establishment of support groups be instituted. The groups need assistance with some human and financial resources.
3. Research be conducted to develop evidence-based rehabilitation programmes for nyaope use.

## Conclusion

The study highlighted the difficult consequences of the family members of nyaope users, which include family conflicts, financial distress, and mental difficulties. Together, these consequences contribute to social, mental, and financial difficulties of family members of nyaope users, which are often not even acknowledged as needs and the limited services are directed at the nyaope user. The need for the development, implementation, and monitoring of formal interventions for families emanate from the findings of this study. Because nyaope addiction is difficult to resolve, these families are subjected to long-term societal and mental challenges, which extends the impact of nyaope use from the user to several members of his or her family. The Department of Social Development needs to form partnerships with NGOs, to extend its footprint as the number of nyaope users increase in communities.
